# Changes in Serum Levels of Myokines and Wnt-Antagonists after an Ultramarathon Race

**DOI:** 10.1371/journal.pone.0132478

**Published:** 2015-07-06

**Authors:** Katharina Kerschan-Schindl, Markus M. Thalmann, Elisabeth Weiss, Maria Tsironi, Ursula Föger-Samwald, Johann Meinhart, Katerina Skenderi, Peter Pietschmann

**Affiliations:** 1 Department of Physical Medicine and Rehabilitation, Medical University of Vienna, Vienna, Austria; 2 Department of Cardiovascular Surgery, Hospital Hietzing, Vienna, Austria; 3 Department of Pathophysiology and Allergy Research, Center for Pathophysiology, Infectiology and Immunology, Medical University of Vienna, Vienna, Austria; 4 School of Nursing, University of Peloponnese, Sparta, Greece; 5 Karl Landsteiner Institute for Cardiovascular Surgical Research, Vienna, Austria; 6 Department of Nutrition and Dietetics, Harokopio University, Athens, Greece; University of Birmingham, UNITED KINGDOM

## Abstract

**Background:**

Regular physical activities have a positive effect on the muscular skeletal system but overstrenuous exercise may be different. Transiently suppressed bone formation and increased bone resorption after participation in a 246-km ultradistance race has been demonstrated.

**Purpose:**

The aim of this study was to analyze effects of the Spartathlon race on novel musculoskeletal markers.

**Methods:**

Venous blood samples were obtained before and immediately after the race from 19 participants of the Spartathlon. From 9 runners who were available 3 days after the start blood was drawn for a third time. Serum levels of myostatin, an inhibitor of myogenic differentiation, and its opponent follistatin as well as sclerostin and dickkopf-1, both of them inhibitors of the wnt signaling pathway, and markers of bone turnover were determined.

**Results:**

Serum levels of myostatin were significantly higher after the race. Serum follistatin only showed a transient increase. Sclerostin levels did not significantly differ before and after the race, whereas dickkopf-1 levels were significantly decreased. At follow-up a decrement of sclerostin and dickkopf-1 levels was seen. Serum cathepsin K levels did not change.

**Conclusion:**

The increase of serum levels of myostatin appears to reflect muscle catabolic processes induced by overstrenuous exercise. After the short-term uncoupling of bone turnover participation in an ultradistance race seems to initiate a long-term positive effect on bone indicated by the low-level inhibition of the Wnt/β-catenin signaling pathway.

## Introduction

Regular physical activities have positive effects on the muscular skeletal system. It has been known for a long time that especially strength training increases muscle mass and that cyclic bone loading is essential for bone homeostasis. The regulating mechanisms are activation of several signaling pathways.

Such endocrine factors are members of the transforming growth factor-ß (TGF-ß) superfamily like myostatin which is also known as growth differentiating factor-ß (GDF-8). It is a negative regulator of skeletal muscle growth [1]. An increase in myostatin gene expression has been reported in muscular wasting [2] and administration of myostatin to adult mice induces profound muscle loss analogous to that seen in human cachexia syndromes [3]. Follistatin, another member of the TGF-ß superfamily, prevents myostatin from binding to its receptor, thereby neutralizing it [4]. Sclerostin (mainly produced by osteocytes) and Dickkopf 1 (Dkk-1; expressed within osteoblasts and osteocytes) are antagonists of the wingless-type mouse mammary tumor virus integration site (Wnt) pathway which is the key pathway for the activation of osteoblasts (for review see [5]). Cathepsin K is a member of the papain family of cysteine proteases and is mainly expressed by activated osteoclasts. Because of its ability to degrade type I collagen it plays a major role in bone resorption [6].

Pro- and anti-inflammatory cytokines as well as myokines are known to change during or after physical activity [7]. For instance, endurance exercise is usually characterized by a decrease in myostatin expression and several studies have shown that resistance exercise induces a decrease of skeletal muscle myostatin mRNA expression [8]. Overstrenous and prolonged exercise may be different. It seems to have some negative effects, inducing inflammation and uncoupling of bone turnover [9]. In all studies investigating participants of the Spartathlon race—a foot race of 246 km—acute inflammatory tissue injury was shown after the run [10–12]. The same was found for other overstrenous physical activities, a triathlon and the Ironman race [13, 14].

Thus, on one hand there exist data on the positive effect of physical activity on the production of several endocrine factors important for homeostasis of muscle and bone metabolism; on the other hand there exist data of overstrenous exercise leading to acute phase reaction, tissue injury, and an increase in bone turnover. In previous work we demonstrated that participation in the Spartathlon led to asymptomatic rhabdomyolysis [15] and transiently suppressed bone formation and increased bone resorption [9]. However, we do not know very much about the effect of participation in such an overstrenous ultradistance foot race on musculoskeletal markers like myostatin, follistatin, sclerostin, Dkk1, and cathepsin K; to analyze serum levels of these myokines and osteokines in Spartathlon participants was the aim of this study.

## Methods

### Study population

All participants of the Spartathlon race were invited to take part in this study. No specific inclusion or exclusion criteria for the study were defined because candidates of such a sport event are supposed to be healthy. There are very stringent prerequisites for participants of the Spartathlon. Athletes are only allowed to start if they have managed another ultradistance run before. Competitors’ usual amount of training encompasses about 100 km during winter time and more than 200 km during summer—up to 7000 km per year. The race is an ultramarathon foot race of 246 km distance which takes place once a year. Runners start in Athens and have to reach Sparta within a time limit of 36 hours. Partially the Spartathlon runs over rough tracks and muddy paths, crosses vineyards and olive groves, climbs steep hillsides and, most challenging of all, takes the runners on the 1,200 meter ascent and descent of Mount Parthenio during nighttime. The environmental conditions are also demanding. During the day temperatures are between 27 and 34°C without any shade and during the night they drop to about 10°C; additionally it is often windy. Thus, it is not surprising that the energy consumption amounts 20 000 kcal. Participants are allowed to consume fluids (usually about 35 to 45 liters) and carbohydrate-rich foods without any limitations offered at 75 checkpoints along the whole distance. The study was approved by the institutional review board of Harokopio University, Athens. All runners were informed about the procedures and purposes of the study and gave their written consent prior to participation in the study.

### Biochemistry

Venous blood samples were drawn from an antecubital vein three times: on the day before the start, within 15 minutes after the end of the race, and three days after the start of the race. Samples one and three were collected in the morning to eliminate diurnal variations in serum levels of the bone-specific biochemical variables. Thus, only the time of collecting sample two differed depending on the time of the day the individual runner completed the race.

Serum was separated from whole blood by centrifugation and then immediately frozen and stored at -70 degrees until assayed. Using high amounts of dry ice samples were continuously kept at -70 degrees during shipment to the Medical University of Vienna where biochemical analyses were performed. All samples were handled in a single batch run. The following musculoskeletal markers were investigated: myostatin (colorimetric competitive immunoassay, Immundiagnostik, Bensheim, Germany, limit of blank LoB: 0.370 ng/ml, (B0 + 1.645 SD); intra-assay coefficient of variation: <12%, inter-assay coefficient of variation: <14%, according to manufacturer’s data), follistatin (colorimetric sandwich immunoassay, R&D Systems, Minneapolis, USA, MDD range 0.005–0.068 ng/mL; mean MDD 0.016 ng/mL; intra-assay coefficient of variation: <3%, inter-assay coefficient of variation: <10%, according to manufacturer’s data), sclerostin (BI-20492, colorimetric sandwich immunoassays, Biomedica, Vienna, Austria; detection limit: 3.2 pmol/l (0 pmol/l + 3 SD); intra-assay coefficient of variation: ≤7%, inter-assay coefficient of variation: ≤10%, according to manufacturer’s data), dickkopf (Dkk) 1 (BI-20412, colorimetric sandwich immunoassays, Biomedica, Vienna, Austria; detection limit: 0.38 pmol/l (0 pmol/l + 3 SD); intra-assay coefficient of variation: ≤8.0%, inter-assay coefficient of variation: ≤12.0%, according to manufacturer’s data), and cathepsin K (BI-20432, colorimetric sandwich immunoassays, Biomedica, Vienna, Austria; detection limit: 1.1 pmol/l; intra-assay coefficient of variation: ≤6.0%, inter-assay coefficient of variation: ≤8.0%, according to manufacturer’s data). Additionally, two bone turnover markers were studied: procollagen type 1 N-terminal propeptide (P1NP; IDS-iSYS Multi-Discipline Automated Analyzer, Immunodiagnostic Systems Ltd. England, detection limit: 2 ng/mL; intra-assay coefficient of variation: 2,6–3,0%, inter-assay coefficient of variation: 4,2–5,3%) and cross-linked-C-telopeptide of type I collagen (ELISA, IDS-iSYS Multi-Discipline Automated Analyzer, Immunodiagnostic Systems Ltd. England sandwich; detection limit: 0,033 ng/ml; intra-assay coefficient of variation: 2,1–4,9%, inter-assay coefficient of variation: 4,7–8,8%).

### Statistical analysis

Data are presented as medians and quartiles. Pre- and post-race serum levels of the biochemical markers were compared with each other by Wilcoxon’s signed rank test. The Friedman two way analysis of variance by Ranks was used to detect potential differences in the serum parameters between the three time points of 9 runners. Serum levels of myostatin, follistatin, sclerostin, and Dkk-1 are given in Box-Whiskers Plots. Statistical significance was set at p-values less than 0.05. Statistical analyses was done using the software packages GraphPad Prism 5 (Prism 5 für Windows, Version 5.00, 2007) and SPSS Statistics V21 (SPSS Inc., Chicago, IL, USA, 2012).

## Results

Seventy-two participants of 310 starting runners managed to reach the finish line in Sparta within the time limit. Nineteen of them—18 men and one woman—had blood taken the day before they started and immediately after the race. Some of our study participants left Greece before the last blood collection. Thus, blood samples at all three time points were taken from nine runners (8 males, 1 female). The median age of all 19 runners was 45 years [41; 48] and it took them 34 h and 03 min [32 h and 29 min; 35 h and 03 min] to reach Sparta.

All participants’ serum levels of the myo- and osteokines before and immediately after the race are shown in Table 1. A significant increase was detected for myostatin but an even four-fold higher increase of serum follistatin was seen. Comparing post-race serum levels of Dkk-1 with pre-race values showed a small but significant reduction. P1NP was reduced whereas CTX was increased after the run.

**Table 1:** Serum levels of myostatin, follistatin, sclerostin, DKK-1, P1NP, CTX, and cathepsin K on the day before the start of the race as well as within 15 minutes after finishing the race; median [quartiles].

	Before startn = 19	After finishn = 19	P value
Myostatin (ng/ml)	23.73 [21.16; 28.28]	26.73 [21.22; 31.68]	p = 0.0364
Follistatin (pg/ml)	300.8 [236.4; 831.5]	1211 [849.1; 2174]	p = 0.0002
Sclerostin (pmol/l)	29.15 [21.22; 43.51]	27.75 [24.77; 51.66]	n.s.
Dkk-1 (pmol/l)	38.68 [18.76; 48.07]	38.14 [12.73; 46.07]	p = 0.0364
P1NP (ng/ml)	54.37 [38.69; 67.17]	41.14 [26.27; 47.31]	p = 0.0004
CTX (ng/ml)	0.299 [0.216; 0.425]	0.542 [0.309; 0.767]	p = 0.0024
Cathepsin K (pmol/l)	1.8 [0.69; 4.23]	1.4 [0.16; 6.69]	n.s.

**Fig 1:**
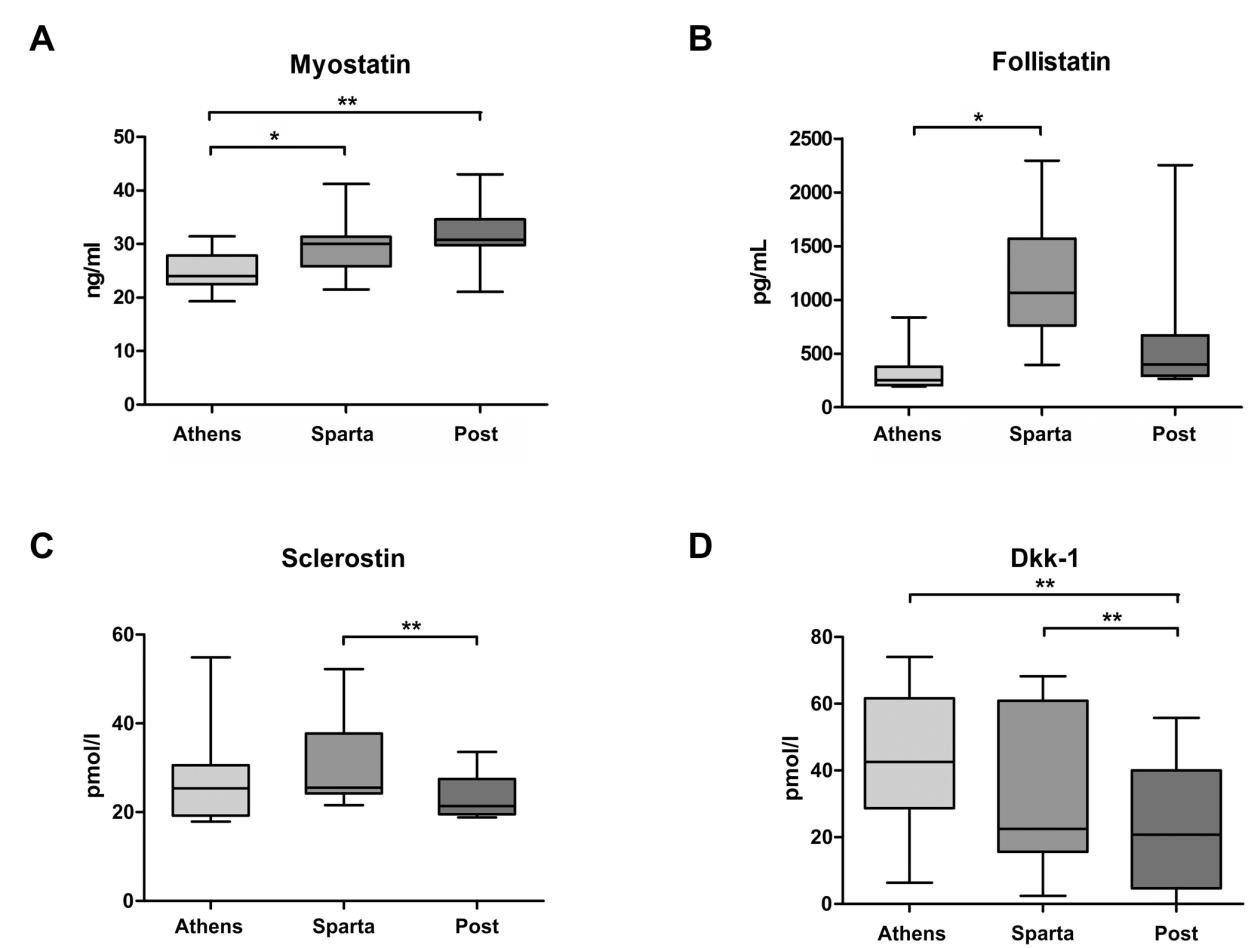
Serum levels of myostatin (A), follistatin (B), sclerostin (C), and Dkk-1 (D) before the run (Athens), immediately after the run (Sparta), and three days after the start of the race (post).

Similar to the whole group of participants, in the group of nine runners with three blood samples taken, serum levels of myostatin and follistatin were higher when reaching Sparta compared to before the race (Fig 1). During the follow-up, myostatin levels remained more or less unchanged. Serum follistatin decreased again; it did not show a significant change 3 days after the start of the race compared to the time point of the first biochemical analysis. Boxplots of serum sclerostin and Dkk-1 for all measurements of the nine participants are also given in Fig 1. In contrast to the whole group of study participants, in the group of nine runners with three blood samples taken, the serum levels of Dkk-1 from pre- to post race did not reach statistical significance. Nevertheless, Dkk1 values three days after the race were significantly lower than at the other two time points. Both inhibitors of the Wnt signaling pathway significantly decreased from immediately after the race to the last measurement.

The bone formation marker P1NP decreased significantly after the race (p = 0.003) and showed a trend to increase again thereafter (p = 0.096). Contrary, serum levels of CTX showed an opposite pattern: There was a trend to increase post-race compared to pre-race (p = 0.096), CTX slightly decreased again in the post-race period (p = 0.739). The median of serum levels of cathepsin K decreased after completion of the Spartathlon (p = 0.034); changes in the follow-up period were nonsignificant ((p = 0.157; Table 2).

**Table 2:** Serum levels of P1NP, CTX, and cathepsin K on the day before the start of the race, within 15 minutes after finishing the race, and three days after the start; median [quartiles].

	Before startn = 9	After finishn = 9	3 days after startn = 9
P1NP	54.37 [41.27 77.56]	41.14 [27.79; 47.25]^a^	46.57 [39.01; 61.12]
CTX	0.342 [0.235; 0.478]	0.613 [0.332; 1.119]	0.55 [0.255; 0.692]
Cathepsin K	2.71 ([0.43; 4.57]	1.73 [0.9; 6.72]^a^	2.010 [0.145; 3.595]

^a^: significant change after finish compared to before the start.

## Discussion

This study identifies the TGF-ß member myostatin as a marker of the muscle catabolic process induced by participation in an ultradistance run of 246 km. It also shows that the short term uncoupling of bone metabolism is followed by a bone-anabolic effect. Serum levels of myostatin increased in our study from pre-race to post-race. Results of previous studies investigating changes of myostatin following physical activities are controversial. Evidence exists that myostatin mRNA is down regulated by endurance as well as resistance exercise. Except for one small study which did not detect any significant changes in myostatin mRNA values after any kind of exercise [16] all other studies showed a decrease in myostatin expression associated with endurance exercise [8]. However, the amount of exercise in these investigations was much lower compared to our study. Several studies showed an association between strength training and a decrease in skeletal muscle mRNA whereas a few studies could not detect any change in myostatin mRNA-expression following strength training (for review see [8]). In contrast to mRNA levels, plasma myostatin protein concentrations were investigated more seldom. They have been shown to increase in two studies of one study group [17, 18] and to decrease in another [19]. However, important factors influencing myostatin expression seem to be duration [20] and intensity of the physical activity. A recent experimental study [21] showed that overreaching leads to myostatin up regulation and there is no doubt that a 246 km run is overstrenous. However, when interpreting the increase of serum myostatin in our study’s participants one has to keep in mind that its opponent follistatin showed a four-fold rise immediately after the race but did not show a significant change 3 days after the start of the race compared to the time point of the first biochemical analysis. The immediate increase of follistatin after exercise is in line with some previous studies. Strength training [17, 22] as well as endurance training [23, 24] showed increases in follistatin-like related gene expression in experimental as well as non-experimental designs. No changes in follistatin mRNA or follistatin-like related gene attributable to strength training were published a few times [20, 25, 26] but one of these study groups found an increase of follistatin-like 3 following exercise in a later investigation [27]. Anyway, this is the first investigation on the effect of such an extended physical performance on serum levels of follistatin. We have shown previously that participation in an ultradistance race induces muscle damage immediately after the run [15] but this study gives more details on muscle metabolism. The negative regulator of skeletal muscle growth, myostatin was increased at both time points after the run; serum follistatin was transiently raised but at the final investigation not significantly different from baseline. We thus cannot exclude that the increase of follistatin immediately after the race at least in part was due to hemoconcentration. The rise of serum levels of myostatin is in line with two previous studies [17, 18]. We speculate that it would be a reflection of catabolic processes induced by cortisol or proinflammatory cytokines which are known to increase during a very stressful event.

Evidence exists that the key pathway of activation of osteoblasts is changed through exercise. Serum sclerostin levels have been shown to be higher in physically active individuals compared to sedentary controls [28–30] but participation in this ultradistance race did not alter serum sclerostin levels immediately after the race. These results are in line with an investigation on mechanical loading over a 1-year period of postmenopausal women which did not lead to a significant change in serum sclerostin levels [31]. However, serum sclerostin levels decreased during the follow-up period. Serum levels of Dkk-1, another important regulator of the Wnt signaling pathway, were reduced when finishing the run and decreased further thereafter. So far, there exists only one study investigating the effect of physical activity on serum levels of Dkk-1 –an experimental study. Its results showing that sedentary rats have higher protein levels of Dkk-1 than exercising animals [32] are in line with our investigation. The decreases in sclerostin and Dkk-1 seem to be an expression of the induction of an anabolic process for bone. Nevertheless, Dkk-1 was a more reliable marker of bone metabolism following extremely strenuous physical activity. More osteoblasts will be evolved because of the reduced inhibition of the Wnt signaling pathway. That fits well with the general knowledge of an anabolic effect of physical activities. In the short-term however, the increase in the serum level of the bone resorption marker CTX and the decrease in the bone formation marker P1NP are an expression of transient uncoupling of bone metabolism which is in accordance with our previous work [17] and has already been suggested previously [33–35]. This uncoupling may be induced by targeted remodeling trying to repair the running induced microdamage in bone. Serum levels of cathepsin K did not show an increase in bone resorption in this study. They may respond delayed in time because in a previous experimental study of 18 months duration serum cathepsin K levels tended to be higher in running compared to sedentary rats [36].

It is well-known that regular bone loading is essential for bone integrity but it has been shown in several studies that the dosage may be a key point. Running distance was negatively correlated with lumbar spine bone mineral density in male and female runners; running distances were greater in subjects with lower bone mineral density– 82 versus 75 km/week [37]. Our previous work [9] as well as this study’s results revealing an uncoupling of bone turnover in the first two days after an ultradistance run of 246-km are in line with this knowledge. However, the new musculoskeletal parameters assessed in this study seem to modify our understanding and suggest that overstrenous work-out in the short term is negative but initiates a long-time anabolic effect. It is difficult to quantify this positive effect. In clinical praxis, the diagnosis of osteoporosis and monitoring of the efficacy of therapy are based upon the measurement of bone mineral density. However, this surrogate parameter has its flaws because bone strength also depends on other parameters like bone size, bone quality, architecture and vitality of osteocytes. Broadband ultrasound attenuation (BUA) is no standard for the diagnosis of osteoporosis but it helps to assess aspects of bone quality and may therefore be a good research tool to quantify these effects. Marathon running has been shown to ameliorate the age-associated decline in BUA [38]. Moreover, several parameters of bone geometry were better in sprinters’ than long-distance runners’ tibiae in an investigation that compared track and field master athletes of different disciplines [39]. A possible explanation for this observation could be that ground reaction forces and bone strains as an anabolic stimulus increase with running speed.

A limitation of the study is the relatively small sample size. However, relatively few subjects take part in such an ultradistance run, and the number of subjects reaching the finish line in time is even smaller. In order to account for potential diurnal variations in biochemical markers a second post-race blood sample in the morning after the completion of the race was taken. During this time span runners had the opportunity to correct their fluid balance. Thus, blood sample three appears to be the most relevant for interpretation of alterations in muscle and bone metabolism. Of course, it would have also been very interesting to investigate aspects of the runners’ bone quality but because of a lack of feasibility that was not possible.

## Conclusion

The increase of serum levels of myostatin appears to reflect muscle catabolic processes induced by overstrenous exercise. The decline of serum sclerostin and Dkk-1 means a low-level inhibition of the Wnt/β-catenin signaling pathway after the exercise, thereby increasing the differentiation of bone forming cells. Thus, participation in an ultradistance race leads to uncoupling of bone turnover in the short-term but such an overstrenous exercise also seems to initiate a long-term positive effect on bone.

## Supporting Information

S1 Fig Serum levels of myostatin (A), follistatin (B), sclerostin (C), and Dkk-1 (D) before the run (Athen), immediately after the run (Sparta), and three days after the start of the race (post); *: p = 0.02; **: p = 0.003 (DOCX)
