# Supplementary material for: Changes in Serum Levels of Myokines and Wnt-Antagonists after an Ultramarathon Race
Source: PLoS One. 2015 Jul 6;10(7):e0132478. doi: 10.1371/journal.pone.0132478 (PMC4493015; doi:10.1371/journal.pone.0132478)
Supplement: S1 Fig — (DOCX) [file pone.0132478.s001.docx]

Dear leadies and gentlemen,

1. & 2. Since the initials MT were listed twice in the Author Contributions section, Markus Thalmann is changed to Markus M Thalmann now. Thus, the author contributions are correct as listed below.

Author Contributions: conceived and designed the experiments: KKS, MMT, UFS, JM, KS, PP

Author Contributions: performed the experiments: MMT, MT, KS

Author Contributions: analyzed the data: KKS, MMT, EW, UFS, JM, KS, PP

Author Contributions: contributed reagents/materials/analysis tools: EW, UFS, PP

Author Contributions: wrote the manuscript: KKS, UFS, PP

Funding and competing interests are correct.

3. Affiliation information is added in the byline.

4. Headings are modified; they are not written in all capital letters or all italics.

The in-text citations were carefully checked; they are in ascending numerical order.

5. Tables 1 and 2 are reformatted, they do not contain hard returns any more.

6. Fig. 1 is sized correctly now (190 x 137 cm).

With kind regards,

Katharina Kerschan
